# Liposarcome myxoide sous cutané

**DOI:** 10.11604/pamj.2017.26.162.8494

**Published:** 2017-03-21

**Authors:** Asmâa Naim, Nadia Benchekroune, Zineb Bouchbika, Nezha Taoufiq, Hassan Jouhadi, Souha Sahraoui, Abdelatif Benider

**Affiliations:** 1 CHU Ibn Rochd, Université Mohammed VI des Sciences de la Santé, Hopital Cheikh Khalifa, Casablanca, Maroc; 2 Centre Mohamed VI du Traitement du Cancer, Casablanca, Maroc

**Keywords:** Liposarcome, myxoïde, superficiel, cutané, sous cutané, Liposarcoma, myxoid, superficial, cutaneous, subcutaneous

## Abstract

Bien que les liposarcomes myxoïdes (LSM) soient la forme la plus fréquente des sarcomes des tissus mous chez l’adulte, leur localisation primaire superficielle est rare. Ainsi, on individualise deux formes de sarcomes: superficiel et profond qui sont distincts non seulement par leur siège et leur fréquence mais aussi par leur pronostic qui est relativement meilleur dans les sarcomes superficiels. Nous rapportons une observation d’un liposarcome sous cutané de la cuisse droite. Le diagnostic a été confirmé histologiquement après une année d’évolution de la symptomatologie clinique. La prise en charge a consisté en une exérèse large suivie d’une radiothérapie locorégionale. L’évolution a été marquée par une rémission complète maintenue après un recul de 32 mois. Nous soulignons, par la présente observation que la rareté des sarcomes superficiels peut être source de retard diagnostic, ce qui pourrait compromettre leur pronostic.

## Abstract (en)

Although myxoid liposarcomas (MLS) are the most common type of soft-tissue sarcomas in adults, their primary location in the superficial tissues is rare. So sarcomas are classified as superficial and deep-seated. They are distinct based on their location and their frequency as well as their prognosis which is relatively better in superficial sarcomas. We here report the case of a patient with subcutaneous liposarcoma in the right thigh. The diagnosis was confirmed by histology after clinical symptoms had evolved over 1 year. Treatment involved wide surgical resection followed by locoregional radiation therapy. Patient’s evolution was marked by complete remission maintained after 32 months follow-up. This study highlights that superficial sarcomas are rare and this can cause a delay in diagnosis compromising their prognosis.

## Introduction

Le Liposarcome est une tumeur maligne d’origine mésenchymateuse, c’est le deuxième type histologique le plus fréquent des sarcomes des tissus mous après l’Histiocytome malin fibreux. En effet, il représente à lui seul 14-18% des tumeurs malignes des parties molles [1, 2]. Liposarcomes sont des tumeurs des tissus mous profonds, leur extension aux tissus sous-cutanés à partir d´un plan aponévrotique est non commune. Quant à leur localisation primaire sous cutanée elle est très rare, elle est définie par un siège exclusivement au-dessus du fascia superficiel sans envahissement de l'aponévrose. Le diagnostic de liposarcome est confirmé par la présence de lipoblastes [3]. Nous rapportons une nouvelle observation de liposarcome Myxoïde sous cutané.

## Patient et observation

**Figure 1:**
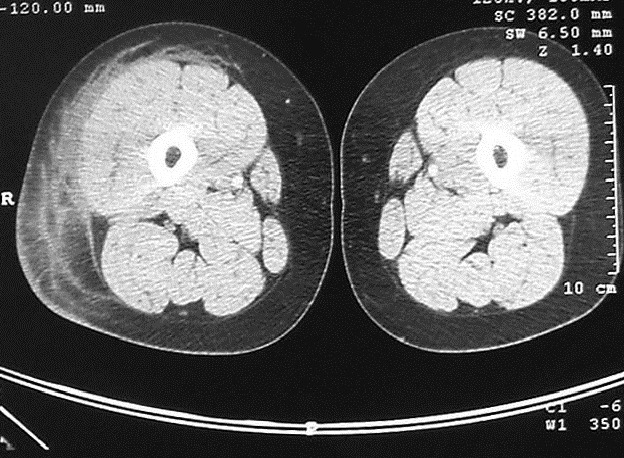
TDM en coupe axiale en faveur au niveau d’une infiltration œdémateuse du tissu graisseux sous cutané de la cuisse droite

**Figure 2:**
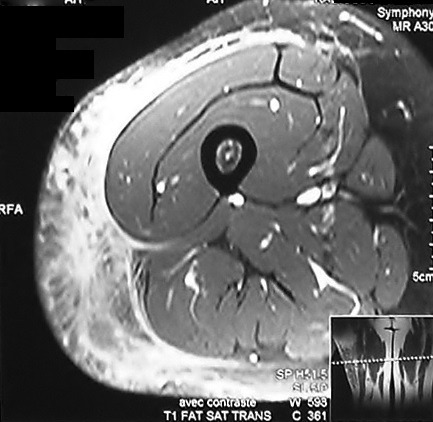
IRM avec saturation de graisse montre une infiltration tumorale des parties molles graisseuses externes de la cuisse droite mal limitée, hypo intense en T1 avec rehaussement hétérogène après injection de Gadolinium

**Figure 3:**
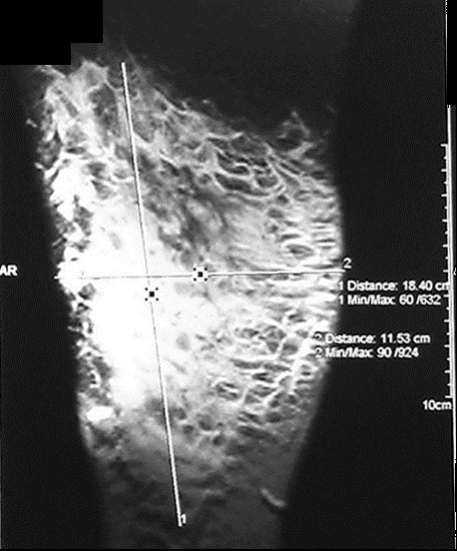
IRM de la cuisse droite objectivant un processus tumoral des parties molles mesurant 184 x 115 mm sans retentissement osseux ou musculaire

Mme B.B âgée de 40 ans, ayant comme antécédent une hépatite B en 1996 et traitée tuberculose ganglionnaire 2001. Présente depuis Septembre 2011 une tuméfaction indolore de la face externe de la cuisse droite. Une Echographie a été faite en octobre 2011 a objectivé une infiltration hématique de la graisse sous cutanée, complété un mois après par un scanner de la cuisse droite qui a montré une infiltration œdémateuse du tissu cellulaire graisseux sous cutané des deux tiers inférieurs de la cuisse droite. L’évolution a été marquée par l’augmentation progressive de la tuméfaction avec apparition de lacis veineux d’où une biopsie a été réalisée en octobre 2012 qui a mis en évidence un aspect compatible avec un Liposarcome Myxoïde. La patiente a été adressée à notre service où un bilan a été réalisé à savoir une IRM de la cuisse droite qui a objectivée une Infiltration tumorale des parties molles graisseuses externes de la cuisse droite, sous forme d’infiltration étendue, mal limitée, hypo intense en T1, hyper intense en T2 ainsi que sur les séquences avec saturation de graisse. Cette lésion est étendue sur 184 x 115 mm sans retentissement osseux ou musculaire par ailleurs. L’injection de Gadolinium entraine un rehaussement hétérogène de cette lésion (Figure 1, Figure 2, Figure 3). Le bilan d’extension à distance était normal. Le dossier a été discuté en RCP est la décision était d’opérer la patiente une exérèse large a été réalisée à l’examen anatomopathologique de la pièce opératoire a retrouvé un foyer Myxoïde de tissu fibreux œdémateux inflammatoire et renfermant de multiples petits foyers mal limités de prolifération tumorale, à stroma Myxoïde, richement vascularisé, avec nombreux vaisseaux à paroi fine ectasiques et congestifs parfois anastomotiques la cellularité est modéré ou marqué par place. Elle est faite d’éléments étoilés, à cytoplasme mal définis et à noyaux hyperchromes avec des atypies modérées. L’index mitotique était modéré avec présence de rares cellules lipoblastiques au sein de la prolifération. Concluant à un Liposarcome Myxoïde grade 2 d’exérèse complète. La patiente a reçu une radiothérapie locorégionale à la dose de 60Gy à raison de 24 fractions de 2.5Gy sur le lit tumoral. L’évolution a été marquée par une rémission clinique et radiologique complète maintenue après un recul de 32 mois.

## Discussion

Le Liposarcome Myxoïde (LSM) représente moins de 10% des sarcomes des tissus mous profonds. Sa localisation primaire superficielle est très rare elle varie de 1-20% de l’ensemble des cas de LSM toutes séries confondues [1, 4]. On distingue deux sous catégories de LSM superficiel: cutané et sous cutané. Le LSM cutané siège au niveau du derme, avec ou sans extension dans l’hypoderme, alors que LSM sous-cutané prend naissance dans l’hypoderme. Contrairement au LSM profond, la forme superficielle est indolente [5, 6]. De même, le LSM superficiel présente une double particularité clinique, d’une part il est potentiellement le type histologique le plus agressif, notamment en cas de présence de cellules rondes. D’autre part, c’est le seul sarcome qui a une prédilection pour des métastases au niveau des parties molles et l’os, souvent bien avant l’atteinte métastatique pulmonaire [4]. Nous rapportons un nouveau cas de LSM primaire sous-cutané des extrémités plus précisément de la cuisse droite. Radiologiquement, le LSM superficiel se présente à l’IRM comme une tumeur hétérogène avec une composante graisseuse en hypersignal T1 et hyposignal T2; la composante Myxoïde est suspectée devant un signal très intense en T2 [7]. Sur le plan anatomo-pathologique le liposarcome est une tumeur maligne mésenchymateuse correspondant à une prolifération de cellules adipocytaires atypiques pouvant ressembler à des adipocytes matures mais le plus souvent constitués d’adipocytes immatures appelés lipoblastes. Le principal diagnostic différentiel du LSM superficiel est le dermato fibrosarcome protubérant (DFSP) dans sa variante Myxoïde qui est également composé de cellules étoilées distribuées de façon lâche dans un stroma Myxoïde. La caractéristique la plus utile afin de le distinguer de LSM superficiel est la disposition très homogène des fibroblastes dans le DFSP en rayons de roue « storiform pattern » [4]. Il a été largement démontré que la prise en charge optimale des LSM repose sur une résection tumorale complète avec marges négatives. Quant à la radiothérapie adjuvante, quoique sa place et ses indications soient bien codifiés dans les formes profondes, son rôle dans LSM superficiel reste moins clair [8]. Dans notre cas, la patiente a bénéficié d’une exérèse complète avec marges saines dont la plus proche à 5cm suivie d’une radiothérapie adjuvante à la dose de 60Gy. L’évolution était favorable avec un contrôle locorégional et à distance maintenu après un recul de 32 mois. Certes, l’évolutivité des Liposarcome varie selon le type histologique, en effet sa variante Myxoïde demeure le principal facteur pronostique défavorable. D´autres paramètres ont été suggérés dans la littérature comme facteurs de mauvais pronostic tel que l´âge avancé, la présence de nécrose, la surexpression du p53 et expression de l’AXL tyrosine kinase [3, 4]. Cependant, la survie à 5 ans des sarcomes superficiels reste relativement meilleur que celle des sarcomes profonds. Cet avantage en terme de Survie Globale a été confirmé par de nombreuses études dont la plus récente est celle de Salas et al. où la Survie Globale à 5 ans était de 80,9% versus 61,4% en faveur des sarcomes superficiels [9, 10]. De même, il a été démontré que la survie sans métastases à 5 ans est meilleure dans les sarcomes superficiels (80.5%) comparativement aux sarcomes avec franchissement du fascia (70%) [9].

## Conclusion

Notre observation souligne la nécessité d’évoquer devant toute masse indolente cutanée ou sous-cutanée de croissance rapide un sarcome superficiel des tissus mous. Ce dernier, représente une catégorie distincte de sarcome ayant un pronostic meilleur sous réserve d’une prise en charge adéquate locorégionale.
